# Telerehabilitation in Low-Resource Settings to Improve Postural Balance in Older Adults: A Non-Inferiority Randomised Controlled Clinical Trial Protocol

**DOI:** 10.3390/ijerph20186726

**Published:** 2023-09-07

**Authors:** Valeska Gatica-Rojas, Ricardo Cartes-Velásquez

**Affiliations:** 1 Telerehabilitation Technology Centre and Neurosciences in Human Movement, Faculty of Health Sciences, Universidad de Talca, Av. Lircay S/N, Talca 3460000, Chile; 2 School of Medicine, Universidad de Concepción, Concepción 4030000, Chile

**Keywords:** COVID-19 pandemic, telerehabilitation, older adults, lockdown, remote

## Abstract

**Background:** Several exercise methods with virtual reality devices have been used in treatments for older adults and patients with neurodegenerative diseases, although the mechanisms continue to be elucidated. The aim of this study is to establish the feasibility and effectiveness of a rehabilitation programme using low-cost virtual reality aimed at improving postural balance in older adults. It also seeks to compare low-cost virtual reality under two delivery modalities, telerehabilitation (TR) in elderly centres and face-to-face (FtF) in rehabilitation centres. **Methods:** The study is set up as a non-inferiority two-arm parallel triple-blind randomised controlled clinical trial. Sixteen persons aged 65 to 75-years-old will be included. Eighteen Wii therapy sessions (25–30 min) will be provided through both FtF (control group, *n* = 8) and TR (exposure group, *n* = 8), both with a Nintendo Wii balance board. Data will be collected at baseline (week 0), during the Wii therapy sessions (weeks 2, 4, and 6), and during the follow-up (weeks 8 and 10). The primary outcome will be the area of centre-of-pressure (CoP) sway; secondary outcomes will be medial–lateral and anterior–posterior velocity and standard deviation of CoP; and tertiary outcomes will be clinical measures: single-leg stand, timed up-and-go tests, Barthel Index, and Tinetti’s scale. Statistical analyses will be performed using SPSS 20.00 for Windows. The trial adheres to the Declaration of Helsinki and the Chilean laws of rights and duties of the patient and research in humans. Ethical approval was obtained from the Ethics Committee of the University of Talca. Written informed consent will be obtained from participants. **Discussion:** In this trial, older adults from a Chilean city with a large rural and underserved population share will be included to test the feasibility and effectiveness of a rehabilitation programme using low-cost VR aimed at improving postural balance to generate evidence to support decision makers generating public health policy. **Trial registration:** Australian New Zeeland Clinical Trials Registration (ACTRN12621001380886).

## 1. Background

One of the most affected motor skills in the elderly population is the loss of postural balance, accentuating the risk of falls and a series of traumatic events that include hip fracture [1]. For several years, many countries in the world promoted the importance of physical exercise in older adults and its beneficial effects for postural balance, functional mobility, and quality of life [2,3,4]. Chile, one of the first Latin American countries to lead the development of public policies and physical activity programmes in communities for the elderly [5], has had to stop these programs because of the COVID-19 pandemic. Among the strategies to stop the advance of COVID-19 has been restricting movement of the general population. In line with this, many countries turned to restrictive policy measures where the freedom of movement of their citizens was limited [6]. Confinement strategies can accentuate the effects of physiological aging in the elderly population, leading the individual to a greater functional decline in their daily activities further deteriorating the loss of postural balance and its consequences [7]. It is well known that during aging cognitive and motor functions may be influenced by peripheral and central degenerative phenomena such as decreased neuroplasticity and sarcopenia [8,9,10]. 

Physical exercise has been widely recognised for its numerous benefits in the older adult population. It enhances neural function, cardiopulmonary performance, physical integration, respiratory muscle performance [11,12,13], postural balance [14,15,16,17], and immune system activity [18,19]. However, besides the benefits of physical exercise [20], an important part of the world population remains physically inactive, especially older adults [21], which has been aggravated by the application of confinement strategies that limit the possibility of carrying out guided physical activity in this population [6]. Therefore, creating home movement strategies for older people is a priority. 

Respiratory diseases that affect the elderly not only affect inspiratory muscles but also negatively affect postural balance in this population, accentuating the risk of falls and their traumatic consequences [22]. These findings are key to consider to prevent the physical sequelae of respiratory diseases, which are accentuated by voluntary or compulsory confinement in this population. Ferraro et al. [22] proved that unsupervised physical inspiratory muscles training at home twice each day for 8 weeks significantly improved not only inspiratory muscle function but also postural balance in older adults. Physical exercise is essential for older adults to keep good postural balance, which helps avoid the diverse physical complications caused by lockdown.

Several exercise methods with virtual reality (VR) devices have been used in treatments for older adults and patients with neurodegenerative diseases [23,24,25,26]. Interfaces have been created to be used in computer or available electronic games that generate communication between the user and the virtual environment through body movements. The main widely known commercial devices are Nintendo Wii and Xbox 360 Kinect [25]. Interventions with these devices are based on exercise execution where the individual receives sensory stimuli (extrinsic feedback), mainly visual information, to increase the amount of input to allow motor self-adjustments. The use of these technologies seems to improve postural balance of older people, neurological and stroke patients, and patients with Parkinson’s disease [23,24,25,26,27]. Although the mechanisms still remains unclear, VR rehabilitation therapy shows a positive tendency to improve mental and physical health [27,28,29,30].

The recognition of VR-based therapies as accessible and low-cost systems primarily applies to developed or high-income countries [31,32]. However, that is not the situation in low- and middle-income countries (LMIC), underserved populations, and rural settings. For example, high-end VR headsets usually cost USD 600–800, not including the computer or console to connect with. In that regard, some researchers and developers are working to create low-cost VR devices focused on rehabilitation [33,34]. However, development and validation usually take many years until a product is released to the market. Thus, low-cost and available VR systems appear as a relevant option to be considered in LMIC in order to expand VR-based rehabilitation therapies. Nintendo Wii has proven to be an efficient and effective system to implement VR-based therapies in many settings [35,36,37,38]. Moreover, used or refurbished Nintendo Wii consoles and balance boards can be purchased online for USD 100–200. 

The benefits of VR-based therapies in older people and other populations have been demonstrated in in-person or face-to-face modalities, where a physiotherapist typically guides the therapy [23,24,25,26]. However, the COVID-19 pandemic has limited the access to those modalities, which spotlights telerehabilitation (TR) as a necessity to maintain rehabilitation therapies. However, the evidence on TR and VR rehabilitation is scarce, a recently published systematic review on this matter found only seven randomised clinical trials and concluded that VR and TR can be used as a prolongation to conventional therapy [25].

The aim for this study is to establish the feasibility and effectiveness of a rehabilitation programme using low-cost VR aimed at improving postural balance in older adults. It also seeks to compare low-cost VR under two delivery modalities, TR in elderly centres and face-to-face (FtF) in rehabilitation centres.

## 2. Method

### 2.1. Design

**Figure 1:**
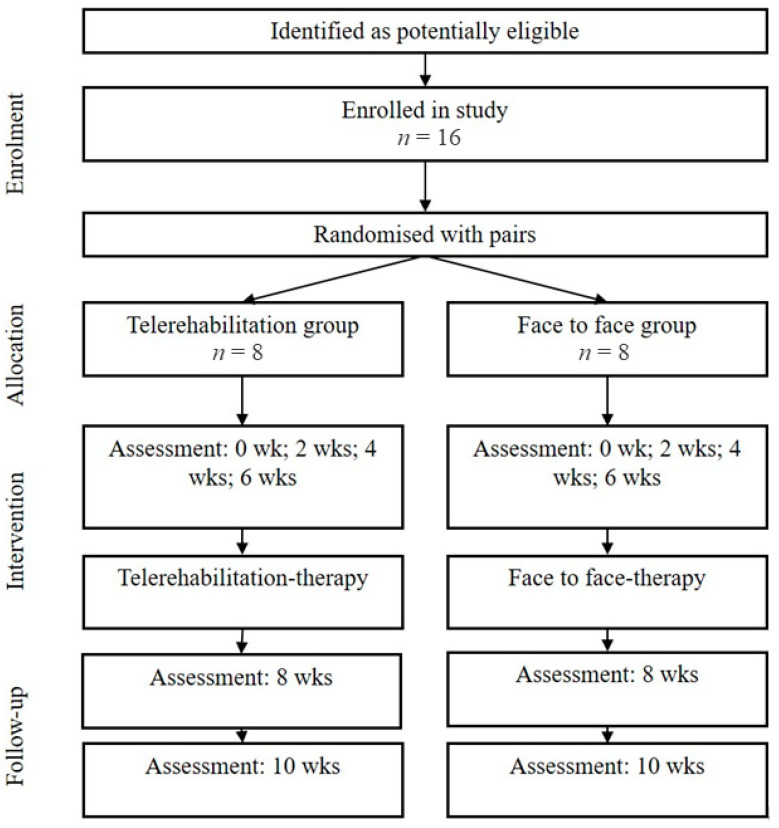
Flow chart according to Standard Protocol Items: Recommendations for Interventional Trials (SPIRIT). Participants, assessments, and interventions in the two groups of the trial.

This is a non-inferiority randomised, triple-blind, controlled clinical trial. Reporting follows Standard Protocol Items: Recommendations for Interventional Trials (SPIRIT [39], see Supplementary File). Figure 1 provides an overview of the trial design. 

### 2.2. Study Setting

This research study examines the impact of low-cost virtual reality in two distinct environments. The first environment is a clinical setting, specifically the Telerehabilitation Technology Centre and Neurosciences in Human Movement at Universidad de Talca in Talca, Chile. In this setting, a low-cost virtual reality intervention is provided in a face-to-face format with a therapist directly interacting with the patient (FtF modality, control group). The second environment is a remote facility, where there is no therapist present, and a low-cost virtual reality intervention is delivered through TR. This will take place in two elderly centres, and the choice of location will be based on the participants’ access to equipment and space. The assessments will be conducted at the facility where the therapy was performed.

### 2.3. Sample Size

The sample size was determined to detect significant changes in the effects of postural balance between FtF and TR groups after six weeks that are clinically relevant. Our proposed difference of equal to or greater than 1.5 units is based on data from previous studies [14,15,16]. A standard deviation of 1 cm^2^, an alpha of 0.05, and 80% statistical power were also considered. A 5% attrition rate was also considered, thus it was determined that a minimum of 8 participants in each group (total sample of 16) would be required. The sample size calculation was performed using GRANMO (Institut Municipal d’investigació Médica, Barcelona, Spain).

### 2.4. Eligibility Criteria

The considered inclusion criteria are: People aged 65 to 75-years-old (women and men), occasional or permanent corrected lens wear, Mini Mental State Examination (MMSE) score of over 24 points, and no falls history in the last 12 months. Exclusion criteria are vestibular impairment and/or access at home to a Nintendo Wii before the intervention. 

### 2.5. Interventions

The objective of this study is to evaluate and contrast the outcomes resulting from the implementation of affordable virtual reality technology across FtF and TR modalities. The low-cost virtual reality intervention entails the utilization of a Nintendo Wii balance board, facilitating the engagement of participants in four exergames.

The FtF group will receive training from a physiotherapist at the telerehabilitation centre of Universidad de Talca, specifically designed for elderly patients. In contrast, the TR group will be trained by an older adult who has undergone six months of training in the rehabilitation program using low-cost virtual reality. These trained older adults will act as therapists for their peers, who belong to two senior citizen centres located near their homes. These centres offer recreational and sports activities for older adults who attend daily. The elderly therapist will receive remote guidance from the telerehabilitation centre at Universidad de Talca, Chile.

FtF and TR groups will undergo a total of 18 sessions, which will be delivered over a period of 6 weeks, with a frequency of 3 times per week (Monday, Wednesday, and Friday) for 25 min per session, followed by four weeks of follow-up for the older adults. For the first 3 weeks, each participant will perform three sets of exercises with manual guidance and verbal instructions. Subsequently, only verbal instructions will be provided by a physiotherapist or elderly therapist. The rehabilitation program has three sets of exercises that improve postural balance in the sagittal, frontal, and transversal planes of motion. The games used in the first two sets of exercises are Snowboard, Penguin Slide, and Super Hula Hoop, while the Yoga game is used in the third set. In the first set of exercises, the older adults will stand in a relaxed position with their arms and hands at their sides. In the second set of exercises, each game will be repeated in a standing position with their hands on their waists. Between the first and second sets of exercises, a one to two minute break will be given, during which the participants will sit on a chair until they have recovered. The third set of exercises involves maintaining a relaxed posture during the Yoga game with their eyes open and then repeating it with their eyes closed.

To encourage adherence, all participants in both the FtF and TR groups will receive telephone reminders. At this stage, no modifications to the intervention protocols will be made throughout the study.

### 2.6. Outcomes

The trial will use posturographic and clinical measures to assess the differences in the effects of FtF and TR groups using low-cost virtual reality interventions for postural control. Posturographic measures assess postural control by measuring the centre-of-pressure sway, including variables such as sway area, trajectories, and velocity in the medial–lateral and anterior–posterior directions [40]. These measures have been shown to be sensitive to measure changes in postural control due to training in various populations, including young adults [41], patients with post-stroke hemiparesis [42], patients with Parkinson’s disease [43], and children with CP [44,45,46,47,48]. Clinical measures will also be used to assess changes in postural performance in the elderly. Participants will be assessed at baseline, the ends of weeks 2, 4, and 6, and at weeks 8 and 10 (post-intervention follow-ups) using both posturographic and clinical measures. Additionally, demographic and clinical characteristics of the study groups will be included.

#### 2.6.1. Primary Outcome

The present investigation will utilize the CoP sway area (CoP_area_) as a dependable and credible indicator of postural control across diverse clinical and nonclinical populations, as demonstrated by previous studies [40,41,42,43,44,45,46,47,48]. CoP_area_ serves as a comprehensive metric of the balance control system’s capacity to sustain a stable upright posture, and higher values of CoP_area_ signify suboptimal balance control.

#### 2.6.2. Secondary Outcome

As for the secondary outcome measures derived from the posturographic evaluations, the standard deviation and velocity of the CoP along the medial–lateral and anterior–posterior axes will be employed. The CoP standard deviation indicates the extent of variation in CoP displacements, while CoP velocity serves as an indicator of the capacity to adapt to postural changes, where higher values are indicative of suboptimal balance control. This is because delayed postural adjustments result in significant CoP displacement, necessitating swift corrections to ensure stability [40].

#### 2.6.3. Clinical Measurements Outcome

This study will use the Tinetti, single-leg stance, and timed up-and-go tests and the Barthel Index as clinical measurements, as described in a recent clinical trial [49]. It is interesting to note that these measures cannot identify fallers in the older adult population but are useful for identifying and tracking balance impairment in this population [50].

### 2.7. Recruitment

The study will recruit older adults from two Senior Citizen Centres in Talca, Chile, where the participants have little to no experience using a Nintendo Wii balance board. The recruitment process will be carried out by a team of experienced health professionals, including a physician and two physical therapists, who will screen potential participants based on the inclusion/exclusion criteria until the required sample size is achieved.

### 2.8. Allocation

Participants will be randomly assigned to either the control (FtF) or experimental group (TR) with 1:1 allocation as per a computer-generated randomization schedule stratified by site and the baseline score of the Action Arm Research Test using permuted blocks of random sizes. For concealing treatment allocation, the block sizes utilised in this study will not be divulged. This statistical approach, tailored to the randomised and block design, has been adopted to ensure the practicality and cost-effectiveness of conducting the clinical trial.

### 2.9. Management

At the beginning of the study, participants will be informed about their random assignment to either the FtF or TR group. To ensure engagement and compliance, older adults in both groups will be regularly asked about the content of the interventions they receive. Data entry will be conducted by an employee outside the research team, who will use separate datasheets to prevent researchers from accessing information about group allocation during data analysis.

The raw data will be stored electronically in encrypted text files, while the calculated and clinical outcome measures will be saved in an encrypted Excel spreadsheet. To ensure data security and prevent loss, backups will be created on a weekly basis.

### 2.10. Data Collection

All measurements will be conducted under identical conditions and by the same assessor at each time point, at a consistent time of day. Posturographic assessments (CoP displacements) for both groups will be obtained utilizing an AMTI OR67 force platform (Watertown, MA, USA). AMTI NetForce software (Watertown, MA, USA) will be utilised for the acquisition of moments and forces at a rate of 100 Hz. Data will be recorded, assigned a code, and saved on a personal computer. CoP variables will be determined using Matlab R2022 (Mathworks Inc., Natick, MA, USA).

All primary and secondary posturographic measures will be evaluated across eight distinct postural tasks, each lasting 60 s, except for the dynamic postural task which will last 30 s. The postural tasks will comprise two static conditions: (i) standing motionless with eyes open, and (ii) standing motionless with eyes closed. In addition, six dynamic conditions will be evaluated: voluntary sway in the mediolateral direction, following a metronome set at 30 Hz ((i) and (ii)) and 60 Hz ((iii) and (iv)), with eyes open and closed for each frequency. Multidirectional sway will also be assessed while playing two different videogames that challenge (v) anterior–posterior postural control (Snowboard, 30 s) and (vi) medial–lateral postural control (Penguin). It is anticipated that all assessments, including clinical evaluations, will be completed within 40 min.

### 2.11. Data Analysis

Statistical analyses will be conducted using SPSS 20.00 for Windows. Descriptive statistics will be utilised to determine the demographic and clinical characteristics of participants in both groups (unpaired *t*-tests and χ^2^ tests). 

The normality and homogeneity of variance of all outcome measures will be assessed using the Shapiro–Wilk and Levene tests, respectively. If the assumptions of normality are met, a t-test will be employed, otherwise, the Mann–Whitney U test will be used to determine differences between the FtF and TR groups. Additionally, if the assumptions of normality are fulfilled, a repeated measures analysis of variance (RM-ANOVA) will be employed, otherwise, Friedman’s test will be used. Post hoc pairwise comparisons will be used to determine the intervention’s effect over time for each group (FtF and TR). Cohen’s d will be used to report effect sizes for the CoP variables. To allow repeated measures analysis, missing data will be adjusted by (1) replacing with the non-missing average values for each variable/week and (2) performing multiple imputations. Both methods will be used to make sure that the results are concordant. For all analyses, a *p*-value ≤ 0.05 will be considered statistically significant. All statistical analyses will be performed using IBM-SPSS 20.0 (SPSS Inc., Armonk, NY, USA).

### 2.12. Data Monitoring

Continuous monitoring will be conducted by the clinical team, overseen by the research team and the Ethics Committee of the University of Talca. In the event of any adverse events, the Ethics Committee of the University of Talca will be immediately notified to determine if any modifications or termination of the trial are necessary.

### 2.13. Patient and Public Involvement

No patients will be involved in the design of the trial.

### 2.14. Ethics

The trial is in accordance with the Declaration of Helsinki and adheres to the Chilean laws regarding the rights and obligations of patients and research involving human subjects. Ethical approval for the trial was granted by the Ethics Committee of the University of Talca (Ref. No. 24-2018). This protocol was registered with the Australian and New Zealand Clinical Trials Registry (ACTRN12621001380886). Written informed consent will be obtained from all participants.

## 3. Discussion

Anticipated findings from this trial suggest that the TR modality will exhibit non-inferior effectiveness to the FtF modality, as evaluated through clinical and posturographic assessments. This expectation draws support from preliminary evidence surrounding the implementation of TR and VR interventions in different settings. According to a recent systematic review, telemonitoring and telerehabilitation were the most used methods of telecare interventions in older populations. The findings from various studies indicated that these interventions had a positive impact on different dimensions of quality of life, particularly in older adults. Evaluating the impact of telecare interventions can provide valuable insights for system developers to improve the effectiveness of current and future telecare technologies to better meet the needs of users, particularly older adults. Future research can focus on evaluating the impact of specific telecare systems on targeted populations using diverse research methodologies. This can help in understanding the effectiveness of telecare interventions in different settings and for different populations and guide the development and implementation of telecare technologies that are tailored to specific user requirements [51]. Similarly, telehealth occupational therapy has been widely utilised for older adults, with a focus on providing convenient occupational assessment and intervention for both occupational therapists and their elderly clients. Telehealth occupational therapy also allows for monitoring of patients’ activities and provides rehabilitation counselling and health education to older adults and their caregivers, resulting in improved home life security and efficacy of occupational therapy interventions. Particularly during the COVID-19 pandemic, telehealth has emerged as a valuable alternative to face-to-face modalities, ensuring continuity of care while minimizing risks of infection [52].

The feasibility and efficacy of telerehabilitation programs seems to be comparable to conventional physiotherapy in terms of functionality level and quality of life, but the evidence is limited to a small corpus of studies. Telerehabilitation has been shown to achieve high levels of patient satisfaction and adherence, with values like those of traditional face-to-face/in-person rehabilitation methods. This suggests that telerehabilitation can be a viable and effective alternative for delivering rehabilitation interventions, with outcomes to comparable those of conventional in-person physiotherapy [53]. 

In the last three years, research on telerehabilitation has risen not only because of the technological improvements and population aging, but also to address rehabilitation needs during lockdowns and to treat post-COVID syndromes as Long COVID [54]. During the COVID-19 pandemic, telerehabilitation has predominantly relied on video and audio calls, utilizing widely accessible technologies and free videoconferencing tools. Based on existing evidence, telerehabilitation is considered feasible and effective in maintaining rehabilitation continuity while minimizing the risk of infection and travel burden during pandemics. However, despite its potential benefits, telerehabilitation is not yet widely utilised in clinical settings, and conclusive findings are currently limited. Nevertheless, telerehabilitation appears to be a viable and safe option for remote delivery of rehabilitation services using readily available mobile technologies, enabling continued care while adhering to social distancing measures imposed by pandemics [55].

Recent research like this trial has found that telerehabilitation, specifically a home-exercise program supervised by physical therapists, has the potential to be as effective as supervised rehabilitation in improving functional outcomes in female patients with patellofemoral pain syndrome [56]. In the case of Long COVID, a virtual rehabilitation programme was highly valued by participants, as the digital delivery enabled self-management of their rehabilitation process. However, there were some barriers to attendance, including challenges related to work/life balance, use of technology, and health inequalities. Additionally, Long COVID, a condition affecting some individuals recovering from COVID-19, was poorly understood by employers, which may have posed challenges for participants in the programme. Overall, despite the positive feedback and benefits of the virtual rehabilitation programme, these barriers and challenges highlight the need for further considerations and support to ensure optimal participation and outcomes for individuals with Long COVID [57]. In a sample of 32 patients with Long COVID, a statistically significant improvement was observed after a 4-week digital physiotherapy practice intervention with an individualised and customised exercise program. The improvement was accompanied by a small to medium effect size, high adherence rates, and values above the minimal clinically important difference. This suggests that the intervention was effective in improving outcomes in this population. However, it is important to note that this conclusion is based on a sample of 32 patients, and further research is needed to validate these findings in larger and more diverse populations [58].

In the case of telerehabilitation focused on older patients, several systems and programmes have been developed over the last years, including some using VR solutions. For example, the use of Virtual Reality Comprehensive Rehabilitation Rooms (VRCRR) in physiotherapeutic treatment for older adults living in a community has been shown to significantly improve their functional performance, particularly in terms of static balance. This innovative approach, using VR technology, offers a viable alternative to traditional physiotherapy methods for enhancing individual functional performance. The self-designed VRCRR solution allows for a tailored physiotherapy programme to be pursued in the comfort of one’s own home environment, making it a promising option for improving physical function in older adults. Therefore, VR technology can be used effectively in physiotherapy management to enhance individual functional performance, especially in terms of static balance, and can be conveniently used at home through a self-designed VRCRR solution. Overall, this suggests that VR can play a valuable role in the rehabilitation of older adults in the community setting [59]. Furthermore, for geriatric patients who have undergone total hip replacement (THR) following a hip fracture, the use of an Internet-based rehabilitation management system has shown to have multiple benefits. Not only does it promote physical rehabilitation, it also plays a positive role in psychological rehabilitation and prevention of complications. This innovative approach offers new ideas and methods for clinical rehabilitation, providing a holistic approach to improve overall patient outcomes. The integration of technology into geriatric rehabilitation can greatly enhance the effectiveness and efficiency of the rehabilitation process, benefiting both the physical and psychological wellbeing of patients recovering from hip fractures and THR. This highlights the potential of Internet-based rehabilitation management systems as a valuable tool in the geriatric rehabilitation field [60].

Moreover, telerehabilitation could be used in frail or cognitively impaired older patients. Telerehabilitation has shown promise in improving the quality of life for older patients with mild cognitive impairment or cognitive frailty, and it can serve as a useful and supportive digital platform for healthcare. Commonly used technologies for telerehabilitation include smartphones or telephones with Internet, television-based assistive integrated technology, mobile applications, and video conferencing. Despite its potential, the utilization of telerehabilitation in managing cognitive frailty among older adults is still limited, and further research is needed to evaluate its feasibility and acceptability. Although telerehabilitation has been implemented among older adults with mild cognitive impairment or cognitive frailty, social support is still necessary to improve adherence and effectiveness. Future research should focus on evaluating the acceptance and existing knowledge of participants towards telerehabilitation to better achieve its intended outcomes. By understanding the perceptions and attitudes of older adults towards telerehabilitation, interventions can be tailored to address potential barriers and enhance acceptance. Additionally, further research can explore the role of social support in facilitating the implementation and effectiveness of telerehabilitation in older adults with cognitive frailty. This can include strategies to provide adequate support, such as caregiver involvement, community resources, and technological assistance, to overcome challenges and ensure successful telerehabilitation outcomes. Overall, continued research and evaluation of telerehabilitation in older adults with cognitive impairments can contribute to optimizing its use as an effective and feasible approach for improving their health and wellbeing [61]. The utilization of cutting-edge exercise equipment designed for rehabilitating frail older adults, in combination with a structured exercise regimen and the incorporation of VR solutions, has demonstrated clear effectiveness. As a result, patients have been able to transition from frailty to pre-frailty stages while experiencing notable improvements in motor and cognitive functions. The integration of modern technology, particularly VR technology, as part of rehabilitation management for older adults with frailty syndrome has proven to be a successful complement to conventional methods. The versatility of VR technology solutions, including their adaptation for home use with remote supervision, has made this innovative rehabilitation approach more appealing to patients, who find it engaging and mentally stimulating. The significant potential of this approach lies in its ability to achieve desirable therapeutic outcomes with enhanced effectiveness [62]. The prospect of incorporating frail older adults into telerehabilitation programs, as outlined in the protocol described herein, represents a significant stride towards extending rehabilitation services to areas where they are most needed. By leveraging telecommunication technologies to deliver rehabilitation interventions remotely, this approach has the potential to overcome geographical and logistical barriers that often limit access to rehabilitation services for older adults in underserved or remote regions. This expansion of rehabilitation coverage to vulnerable populations, such as frail older adults, through telerehabilitation programs has significant implications for addressing healthcare disparities and improving access to care. The integration of telehealth technologies in rehabilitation can facilitate the delivery of tailored interventions, monitoring, and support to individuals in their own homes or local communities, regardless of their geographical location, socioeconomic status, or mental capacity.

## 4. Conclusions

In summary, telerehabilitation has the potential to provide accessible and convenient rehabilitation services, particularly for individuals who may have limited access to traditional in-person rehabilitation, such as older adults or those in remote or underserved areas. Further research and evidence in this area can contribute to the broader adoption and integration of telerehabilitation as a valuable approach in rehabilitation practice. In this trial, older adults from a Chilean city with a large rural and underserved population share will be included to test the feasibility and effectiveness of a rehabilitation programme using low-cost VR aimed at improving postural balance to generate evidence to support decision makers generating public health policy. This aspect warrants significant attention as a substantial portion of the research pertaining to this topic is predominantly conducted in urban areas of high-income countries, which may have socioeconomic conditions that are markedly distinct from those of rural, poor, or underserved areas. As a result, the findings derived from this trial hold particular relevance for settings where the need is most acute. The evidence generated by this study has the potential to be of immense value in informing and guiding interventions in resource-constrained areas, where access to appropriate healthcare resources and services may be limited. 

By addressing the research gap in diverse settings, including those with varying socioeconomic profiles, the findings of this trial can contribute to a more comprehensive and nuanced understanding of the issue, leading to contextually relevant interventions and policies that are tailored to the specific needs of vulnerable populations in underserved areas. This will facilitate the development of more equitable and inclusive approaches to addressing the research question at hand, with a focus on reducing disparities and improving health outcomes in marginalised communities. Consequently, the outcomes of this trial will not only advance scientific knowledge but also have practical implications for policy making and implementation, particularly in settings that are often overlooked or underrepresented in research conducted in more affluent urban areas.

## Data Availability

https://cttn.cl/investigaciones/ (accessed on 25 March 2023).
